# Prof. Siying Peng: caterpillars to butterflies, chasing light in photonics

**DOI:** 10.1038/s41377-025-02111-6

**Published:** 2026-01-04

**Authors:** Ji Wang

**Affiliations:** Westlake University, Hangzhou, Zhejiang China

**Keywords:** Optics and photonics, Optical materials and structures

## Abstract

“To eyelids in the Sepulchre—/ How dumb the Dancer lies—/ While Color’s Revelations break—/ And blaze—the Butterflies!” A renowned American poet, Emily Dickinson’s poem vividly mirrors the journey of women’s growth: No matter how many hardships they encounter in their development or constraints they face, they will eventually break free from their “cocoons” and transform into colorful butterflies radiating “light”. In this issue of “Light People”, Professor Siying Peng is invited to share how the optical properties of butterfly wings have inspired her metamorphosis in the field of photonics.

**Figure:**
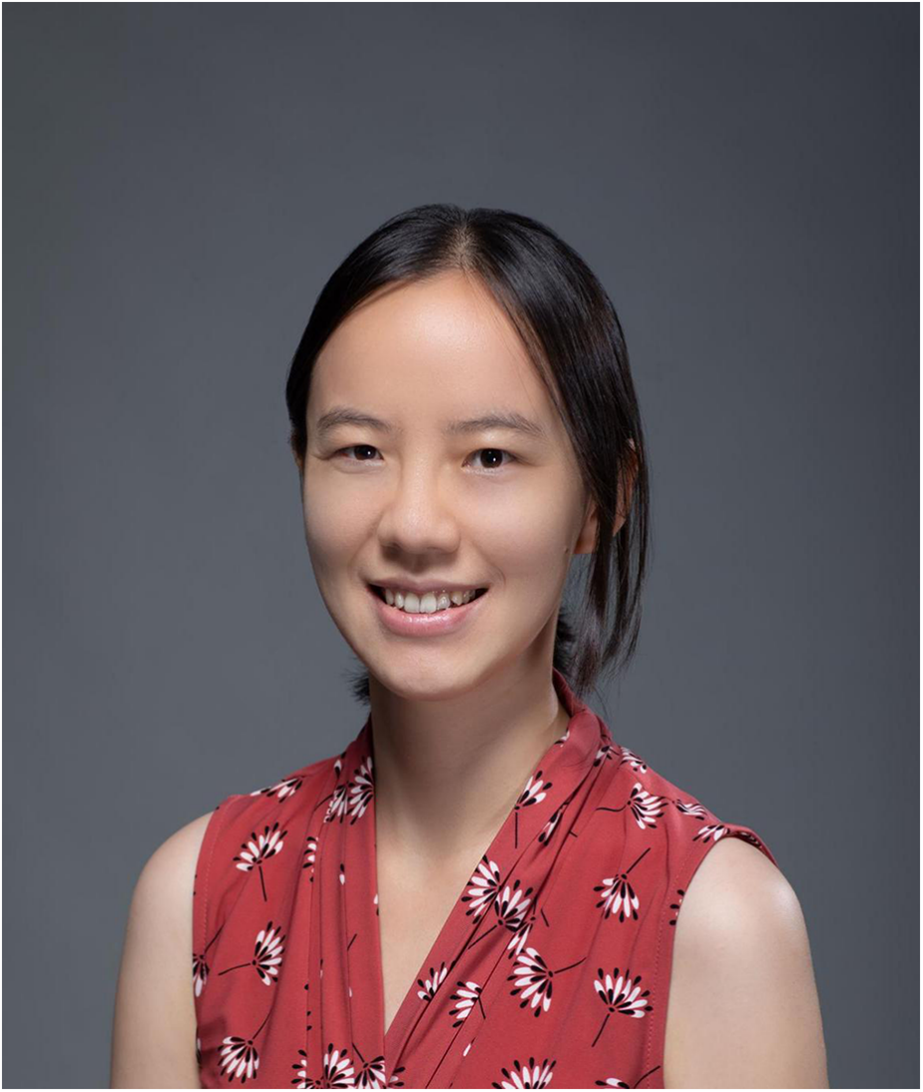

**Short Bio:** Siying Peng is an assistant professor in the Department of Materials Science and Engineering at Westlake University in Hangzhou, China. Siying received her Ph.D. in physics from California Institute of Technology and her B.S. in physics and mathematics from Texas A&M University. She was a GLAM Fellowship postdoctoral researcher at the Geballe Laboratory for Advanced Materials at Stanford University. Her research explores multi-scale light-matter interactions in emerging photonic materials, with broad implications for device-scale technologies. Her research employs in-situ optical transmission electron microscopy to investigate the mechanisms of light-matter interactions, and further translates this understanding into applications such as chiral photonics, excitonic devices, and ultrasensitive imaging.

**1. Could you describe the pivotal moments in your growth that made you feel like breaking through the cocoon and becoming a butterfly, and how these experiences ultimately led you to become a scientist?**

In high school, I took on a personal project to engineer the sound of my alto saxophone because my practice sessions often disturbed my neighbors. I became curious about how much I could reduce the volume of my saxophone through design. At that time, we had no access to Google or AI, so I never managed to do a literature review and had to start thinking from first principles. Drawing on my intuitive understanding of sound propagation within the instrument, I designed a series of silencers varying in shape, length, and material, engineered to fit inside the instrument’s bell.

My grandfather was very supportive of the idea and helped me fabricate the components using materials ranging from wood to foam. I then tested each silencer by playing the instrument and evaluating the sound reduction and tone change by ear. Although the design was rather crude and the test method quite primitive, this experience gave me the first taste of independent discovery. It challenged me to think critically about the physics of sound waves and to use principles of materials engineering to solve a tangible problem.

Looking back, I realize this experience quietly sowed the seeds of my becoming a scientist. Today, my research continues to bridge science and engineering: I work to understand light-matter interactions at the nanoscale and then utilize that insight to design devices with new functions and improved performance.

**Figure:**
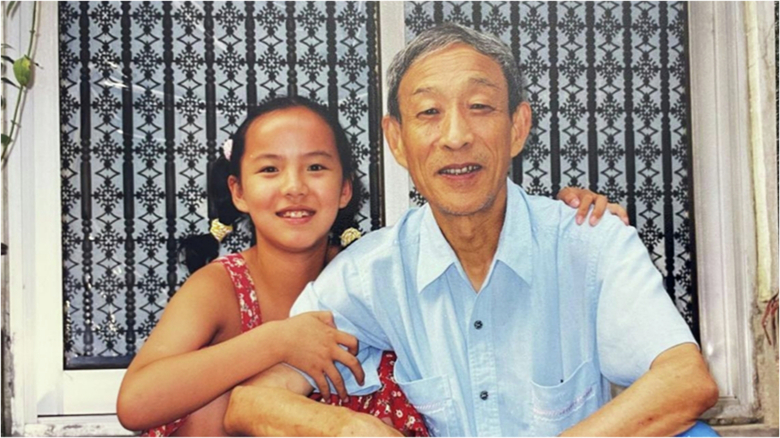
**Siying Peng & Her Grandfather: Childhood Photo**

**2. After you obtained your dual bachelor’s degrees in mathematics and physics from Texas A&M University, what motivated you to pursue a Ph.D. at Caltech under Prof. Harry A. Atwater, a distinguished American physicist and materials scientist with research focuses on nanophotonics and light-matter interactions, solar energy conversion, etc.? How has this choice significantly influenced your research direction?**

I conducted my undergraduate research in Professor Hans Schuessler’s lab, where I received solid training in both optics experiments and electromagnetic wave simulations. I particularly enjoyed the experience because photonics research offers the opportunity to engage in every aspect of a project, from numerical modeling and material synthesis to optical characterization.

Caltech has a cohort of scientists working at the very frontier of photonics, and it also has one of the best cleanroom facilities, so that combination was very attractive to me. Professor Harry Atwater’s group serves as an ideal hub for interdisciplinary research, bringing together doctoral students and postdoctoral researchers across materials science, physics, and chemistry. What impressed me most was that his research group has trained many female scientists in the field, with more than half of the doctoral students being female. Harry is open-minded and strongly supportive of ideas from students, so we got opportunities to explore quite a bit. He possesses a vision that reaches beyond academia and into top-tier industrial transformation, and it has significantly shaped my understanding of science’s role in society. During my time in Harry’s group, I had the opportunity to collaborate with Professor Albert Polman on investigating topological materials with angle-resolved cathodoluminescence spectroscopy. Working with Albert was also a hugely valuable experience for me. I learnt a lot from the way he approaches scientific problems and communicates ideas.

**Figure:**
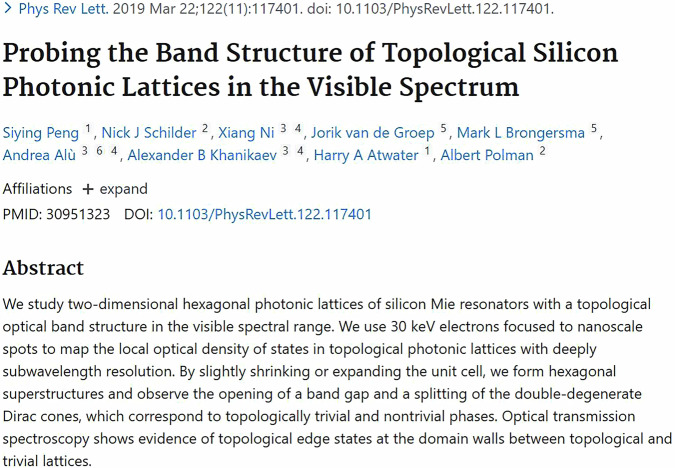
**Siying Peng’s Ph.D. Thesis Work Published in** ***Physical Review Letters***^1^

**3. When did your initial fascination with butterfly wings first cross paths with your research in optics, and what inspired you to explore the optical secrets hidden within these delicate wings?**

Inspiration for my work stems from the unique gyroid structures of butterfly wings—triply periodic body-centered cubic crystals composed of minimal isosurfaces that contain no straight lines. A wide variety of complex derivative structures can be generated from a single gyroid. What’s truly fascinating is that such a remarkable structure can be synthesized naturally within living organisms through a bottom-up process. The geometry alone is captivating.

More intriguing is the structure–property relationship. A single gyroid can support a complete photonic bandgap, allowing it to reflect light entirely at specific wavelengths. This is what gives certain types of butterfly wings their vibrant colors. When a single gyroid combines with its inversion-symmetric counterpart, it forms a double gyroid. When the inversion symmetry of a double gyroid is broken, it can exhibit topological properties. In such structures, light propagating along specific directions is topologically protected and immune to backscattering.

For our project, fabricating gyroid photonic crystals proved to be a major bottleneck. The gyroid is a complex three-dimensional structure. It can only be realized using two-photon lithography. However, we encountered several challenges during the fabrication process: For instance, the photoresist tended to shrink after development; the resulting polymer structures were mechanically unstable; and most critically, the low refractive index of the photoresist led to weak optical responses. With many trials and errors, we were still unable to achieve satisfactory results.

The fabrication issue was eventually resolved through a collaboration with Professor Paul Braun’s group at the University of Illinois at Urbana-Champaign. His group had developed a deposition technique for amorphous silicon with an extremely slow deposition speed, which enabled the conformal coating of gyroids both on their surfaces and within their internal frameworks. This method significantly enhanced the refractive index and optical performance of the structures. With the help of this technique, we successfully fabricated a gyroid photonic crystal that exhibited a complete photonic bandgap.

Even though we ultimately found a viable fabrication solution, I am still deeply fascinated by the natural gyroid structures found in butterfly wings. Nature, through millions of years of evolution, has managed to construct such intricate and high-performance 3D architectures using bottom-up processes. That, to me, is one of the most awe-inspiring miracles of the natural world.

**Figure:**
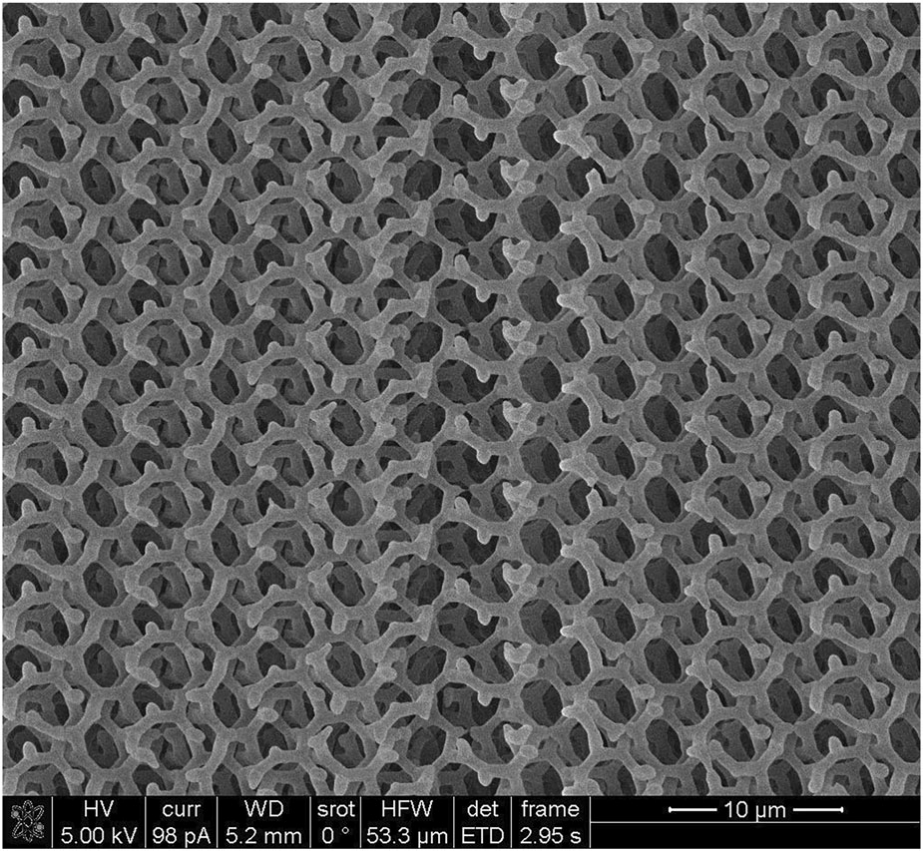
**3D Single Gyroid Photonic Crystals Fabricated by Dr. Siying Peng: An SEM Image**

**4. Besides the color-producing mechanism, are there any other unique optical properties of butterfly wings that you plan to explore?**

The three-dimensional nanostructures found in butterfly wings exhibit fascinating polarization properties. For example, the gyroid structure is inherently chiral. Chirality breaks spatial inversion symmetry, which means that during light–matter interactions, such structures may respond differently to left- and right-handed circularly polarized light. This can give rise to phenomena such as circular dichroism and polarization-selective emission.

Chiral optics has broad implications scientifically and technologically. In fundamental science, it is indispensable in fields like quantum information science and spintronics. For practical applications, it has been used in under-display fingerprint recognition, 3D imaging, biosensing, drug monitoring, etc.

My research group focuses on chiral photonics, where we explore both **intrinsic material chirality**, arising from intrinsic crystal asymmetry, external strain, or external field, and **structural chirality**, which emerges from engineered geometries. We are particularly interested in how light-matter coupling influences chirality. A major research direction is the development of efficient chiral light sources, including circularly polarized light emitters and chiral lasers. We aim to understand the mechanisms by which spin-polarized excitons and optical modes are generated and coupled, and the approach to enhancing these processes through cavity design, strong coupling, and electronic or photonic band engineering. Our goal is to achieve high polarization purity, directional control, and tunability, which are essential for applications in quantum optics, sensing, and display.

**Figure:**
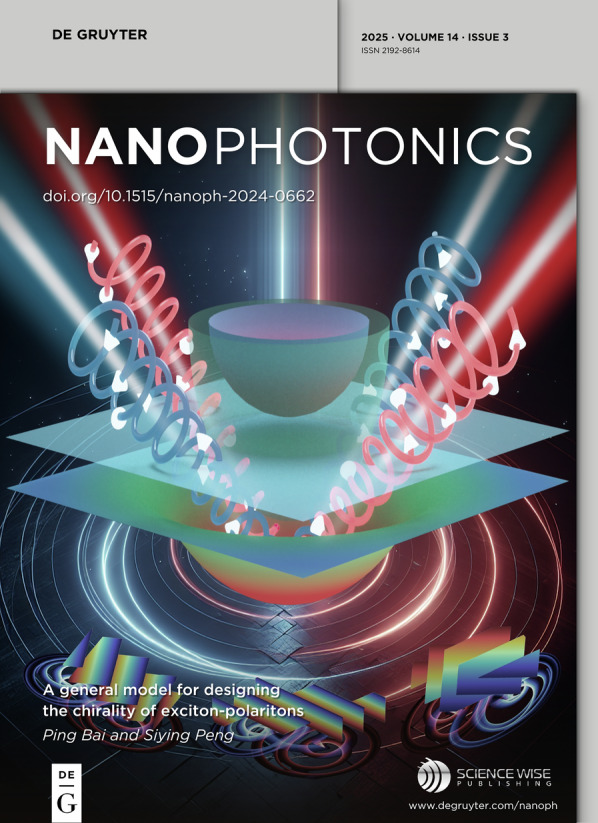
**Prof. Siying Peng’s Team: Cover Paper on Exciton-Polariton Chirality Design in 2025**^2^

**5. How do you think the research on butterfly wings’ structures can contribute to the development of sustainable and environmentally-friendly optical materials?**

Many of the colors found in our everyday products are derived from heavy metal compounds. In contrast, the structural color found in butterfly wings offers a promising, environmentally friendly pathway for developing sustainable optical materials. Structural color originates from the modulation of incident light by subwavelength-scale structures in materials: Certain wavelengths undergo enhanced reflection, transmission, or scattering, while others are suppressed, thereby yielding specific colors.

Structural colors are highly stable and can offer tunable color saturation, making them attractive for a wide range of applications. For instance, structural color has already been demonstrated in labels and anti-counterfeiting applications. Moreover, the nanostructures can be integrated with other functional devices to create multifunctional systems. Examples include colored solar cells, which combine energy harvesting with architectural aesthetics, and colored lithium batteries, which can visually indicate battery status through color change.

**6. How do you think the research on butterfly wings’ structural color mechanism can be translated into industrial applications more effectively?**

There have already been many commercial applications in the field of nanophotonics. One of the most widely used commercial applications is the home pregnancy test, which utilizes the extreme sensitivity of plasmonic resonances of gold nanoparticles to changes in the surrounding refractive index to produce a visible color change in the test strip depending on the presence or absence of the HCG hormone.

In recent years, one of the most promising technologies for commercial applications is metasurfaces. Metasurfaces are subwavelength nanostructures, similar to structures that produce structural colors in butterfly wings, enabling the precise control of the phase, amplitude, and polarization of light. Metasurfaces are compatible with CMOS fabrication processes, which makes them highly advantageous for large-scale manufacturing.

In fact, the latest generation of iPads has already adopted metasurface technology: It uses structured light generated by metasurfaces for facial recognition. I believe this is only the beginning of what nanophotonics can contribute to commercial technologies. As spatial computing in virtual reality applications moves toward lightweight, wearable platforms, the demand for nanophotonics such as metasurfaces will only continue to grow.

**7. In the male-dominated field of photonics, how do you think female scientists can leverage their unique perspectives and approaches to drive innovation and make significant contributions? How have the challenges and chances you’ve faced as a female scientist mirrored the metamorphosis of a butterfly?**

I believe that being a minority can shape a distinct and valuable mindset. Female scientists often learn early in their careers not to rely on conformity or seek external validation, which fosters the development of a strong internal compass. Resilience is another quality that often emerges from navigating such circumstances, and it is especially critical in fundamental scientific research, where progress can be slow and uncertainty is part of the process.

I’ve also found that a heightened sense of observation—particularly toward subtle cues or nuanced patterns (often noted as common traits among women)—is essential for producing high-quality research. This ability lets researchers pick up on small but meaningful details, unusual but critical data points, or unforeseen but breakthrough-driven results, all of which can pave the way for deeper academic insights. More broadly, the capacity to manage multiple tasks effectively—frequently recognized as a strength that many women demonstrate, according to neuroscientific research—is particularly valuable in navigating the overlapping demands of an academic career, such as balancing research, teaching, and administrative responsibilities.

Personally, persistence and patience have been the two qualities that have served me best—they’re exactly what carried me through my own “butterfly metamorphosis” as a female scientist. They allow me to stay committed during long and complex projects, which feel like the cocoon stage—slow, unglamorous, even discouraging, with no “light” in sight; they allow me to find motivation and meaning in the exploration process, even when clear outcomes are not immediately in sight.

**Figure:**
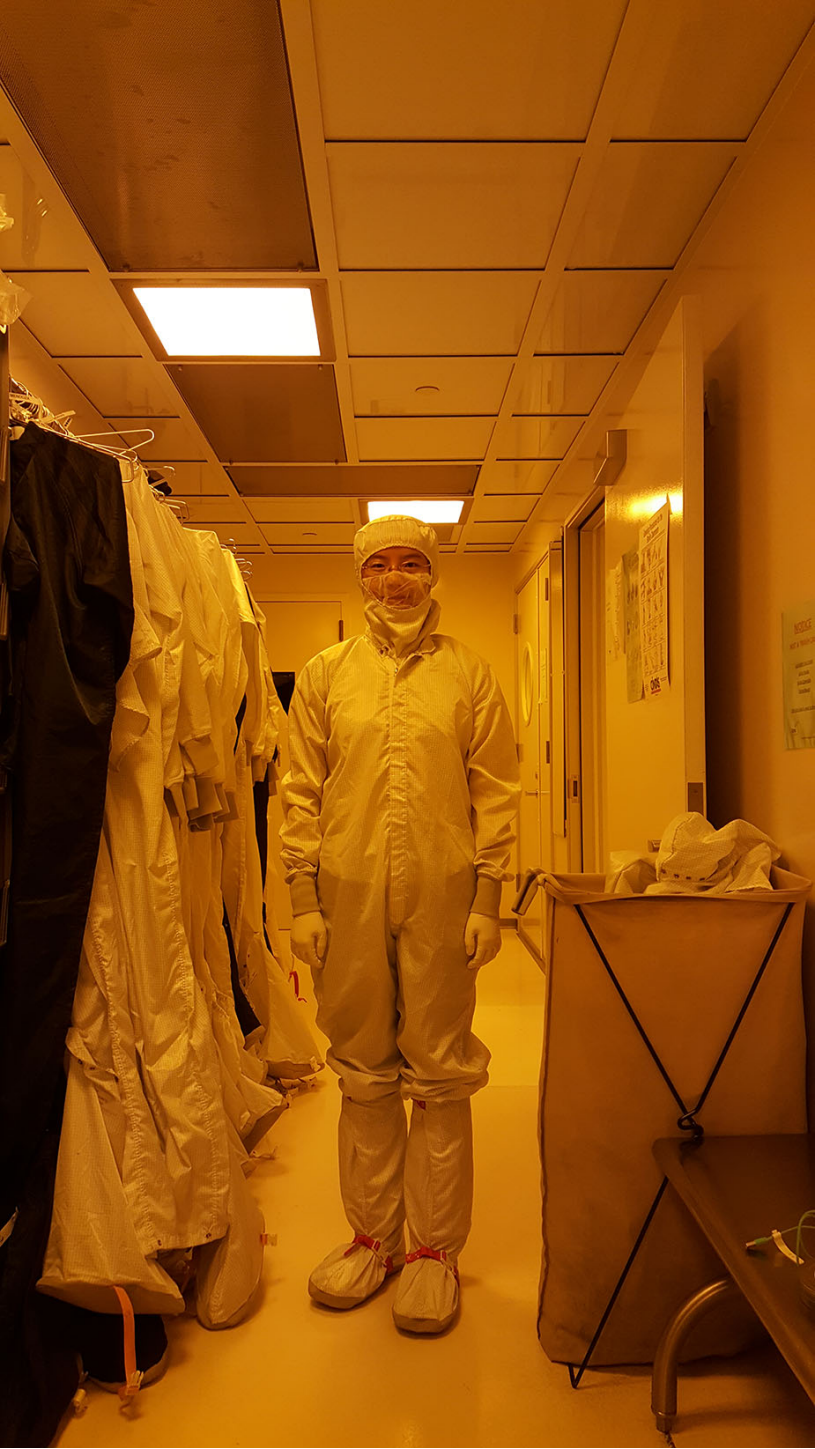
**Dr. Siying Peng Working for the “KNI” (Kavli Nanoscience Institute Laboratory) at Caltech**

**8. In your daily life, how do you balance your scientific career with personal responsibilities? Do you have any hobbies after research work?**

As a female scientist, managing a faculty job that demands nearly 100% of my energy while also making time for family is indeed an incredibly challenging task. It is especially a painful struggle for me after having a child, when time fragments and unpredictability become the norm. In my experience, two things are essential: effective time management and a strong support system.

When it comes to time management, the key is setting clear priorities. It is essential to dedicate sufficient time to important but not urgent tasks, those that truly contribute to long-term progress but can easily be sidelined by immediate demands. Scientific research is a marathon, not a sprint. It requires unwavering motivation and persistence to explore the unknown. At the same time, it demands a temperament that values patience and can find meaning, even joy, in the slow, sometimes frustrating process of discovery.

Family responsibilities undeniably require a great deal of emotional and physical energy. I feel incredibly fortunate to have a supportive partner and an extended family who help share the responsibilities of caring for our child. Their support allows me to be present where it matters most.

Outside the lab, I find tremendous joy in nature. Living in Hangzhou, I am lucky to have access to hiking trails that offer both natural beauty and historical charm. These walks are a source of mental clarity and physical energy for me, which is essential for productivity and creativity. Music is another important part of my life. During my Ph.D. at Caltech, I played alto saxophone in Caltech’s concert band. Music has the magical healing power. It allows me to lose myself completely while I am performing, as if the whole universe were melted into the music. I also enjoyed taking ballet classes, which allowed me to experience the dynamic interplay between movement and music. While at Caltech, I helped organize the Caltech ballet club, where we brought together dance enthusiasts from both the campus and the local community to perform well-known ballet pieces. There were a lot of rewarding and memorable experiences. Now, one of my favorite ways to unwind is simply playing with my daughter. Her boundless curiosity, creativity, and energy are a constant source of strength and inspiration. She reminds me of what it means to be fully present, and to approach each moment with wonder.

**Figure:**
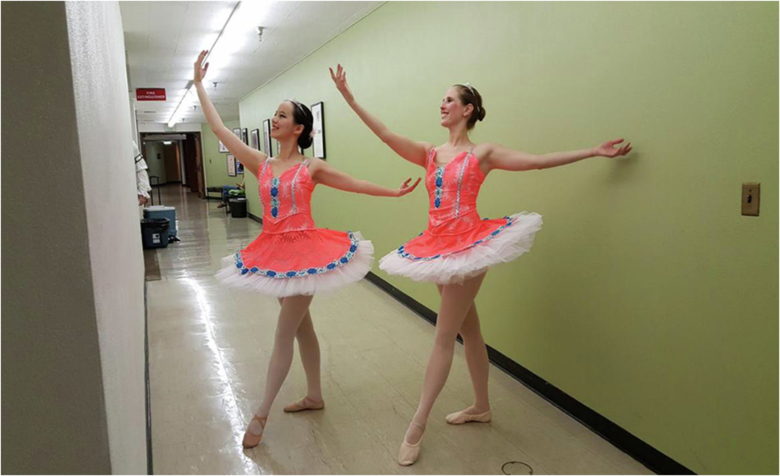
**Dr. Siying Peng Performing in the Ballet** ***The Sleeping Beauty*** **at Caltech**

**9. How does your aphorism “What I cannot create, I do not understand,” quoted from Richard Feynman, a Nobel Laureate in Physics, inspire your research team at Westlake University to move forward in your scientific research regarding nano-optical materials?**

Feynman’s philosophy toward science has always been a deep source of inspiration for me. The quote “What I cannot create, I do not understand,” reflects his belief that true understanding comes from building something from the ground up, not just reading about it, observing it, or memorizing it. He also believed that if you cannot explain a concept intuitively, in a way that someone without a mathematical background can grasp, then you don’t truly understand it yourself.

This is a philosophy I continually remind myself of and also emphasize to my students. In today’s advanced research facilities, it’s easy to generate large volumes of results through automated tools or black-box methods, without fully understanding how they were obtained or what they truly mean. Feynman’s mindset pushes us to dig deeper: Reach the very foundations of a problem, and emerge with a simple, clear message that conveys the essence of what we’ve discovered.

In the age of AI, this becomes even more important. With powerful models at our fingertips, there’s a growing risk of being swept along by data and results without doing the hard intellectual work of understanding the underlying principles. Feynman’s perspective is a crucial reminder: Clarity, intuition, and creation must remain at the heart of scientific inquiry.

**Figure:**
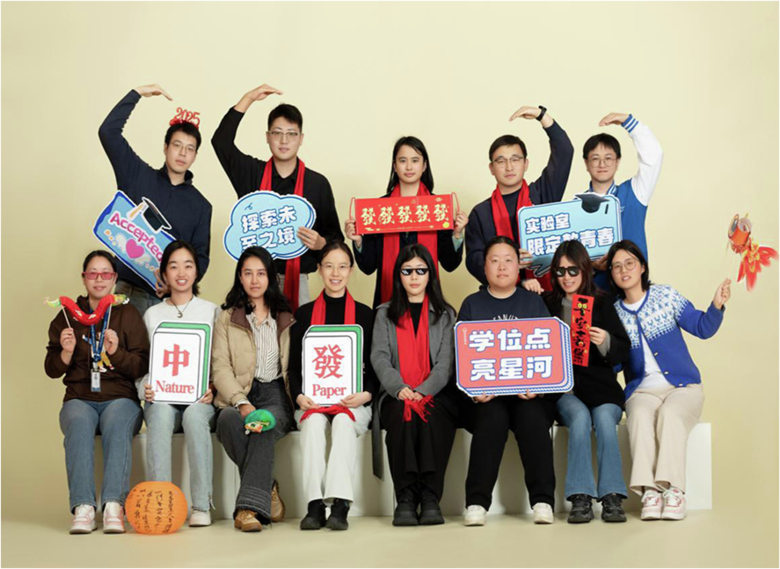
**Prof. Siying Peng’s Research Group at Westlake University**

**10. Looking back on your journey as a female scientist, what advice would you give to young women aspiring to pursue careers in photonics, and how can the beauty and resilience of the cocoon-to-butterfly transformation stand as a source of inspiration for them, guiding their journey through challenges, growth, and self-discovery in this field?**

From my journey of growing into a female scientist, here’s the advice I’d offer young women aspiring to build careers in photonics: When you first develop a fledgling interest in a subject, take the time to shape your unique expressive style and perspectives—that is, to find your own voice. Next, carry this distinct way of thinking and communicating with you as you explore and pinpoint the research area that truly resonates with you. Finally, take the initiative to ensure this voice is heard and embraced: Start by letting your classmates grasp the depth of your thinking, then earn recognition for your ideas from your lab group peers and even within the broader research community, and secure the attention and affirmation your unique insights deserve.

What’s more, this journey of scientific inquiry mirrors the remarkable cocoon-to-butterfly transformation—and it is this transformation’s beauty and resilience that can serve as a powerful source of inspiration for you. Those early, vague sparks of interest in a subject are like the quiet beginnings of a caterpillar’s cocoon; through repeated exploration, they gradually converge into a “core starting point” for your research—the fundamental basis and directional origin that guide all your subsequent work. Scientific research, much like breaking free from a cocoon, is filled with repeated setbacks: Your first paper might feel raw and unpolished; your initial presentation could lack even basic logic. Yet these imperfections are not flaws—they are essential, transformative steps in your growth, just as the struggle of breaking the cocoon is vital for a butterfly’s wings to strengthen. The magic lies in embracing the thrill of “leveling up” through these challenges: That spark of curiosity or discovery becomes the drive to keep refining your work, to persist through difficulties, and to keep learning. As you do this, your research skills will gradually sharpen, your academic taste will mature, and eventually, your own unique voice—your perspective, your ideas, your contributions—will emerge naturally, just as a butterfly emerges gracefully after its struggle.

**Figure:**
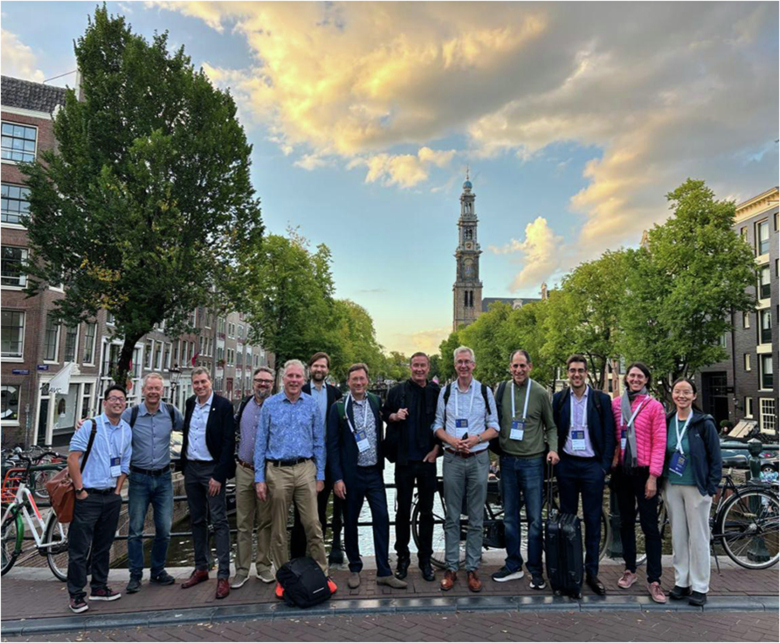
**Prof. Siying Peng Reuniting with Prof. Harry Atwater, Prof. Albert Polman, and Other Friends at Metamaterials 2025**
